# Applying masked autoencoder-based self-supervised learning for high-capability vision transformers of electrocardiographies

**DOI:** 10.1371/journal.pone.0307978

**Published:** 2024-08-14

**Authors:** Shinnosuke Sawano, Satoshi Kodera, Naoto Setoguchi, Kengo Tanabe, Shunichi Kushida, Junji Kanda, Mike Saji, Mamoru Nanasato, Hisataka Maki, Hideo Fujita, Nahoko Kato, Hiroyuki Watanabe, Minami Suzuki, Masao Takahashi, Naoko Sawada, Masao Yamasaki, Masataka Sato, Susumu Katsushika, Hiroki Shinohara, Norifumi Takeda, Katsuhito Fujiu, Masao Daimon, Hiroshi Akazawa, Hiroyuki Morita, Issei Komuro

**Affiliations:** 1 Department of Cardiovascular Medicine, The University of Tokyo Hospital, Tokyo, Japan; 2 Division of Cardiology, Mitsui Memorial Hospital, Tokyo, Japan; 3 Department of Cardiovascular Medicine, Asahi General Hospital, Chiba, Japan; 4 Department of Cardiology, Sakakibara Heart Institute, Tokyo, Japan; 5 Division of Cardiovascular Medicine, Saitama Medical Center, Jichi Medical University, Omiya, Japan; 6 Department of Cardiology, Tokyo Bay Medical Center, Urayasu, Japan; 7 Department of Cardiology, JR General Hospital, Tokyo, Japan; 8 Department of Cardiology, NTT Medical Center Tokyo, Tokyo, Japan; 9 Department of Advanced Cardiology, The University of Tokyo, Tokyo, Japan; 10 Department of Clinical Laboratory, The University of Tokyo Hospital, Tokyo, Japan; Vietnam National University, VIET NAM

## Abstract

The generalization of deep neural network algorithms to a broader population is an important challenge in the medical field. We aimed to apply self-supervised learning using masked autoencoders (MAEs) to improve the performance of the 12-lead electrocardiography (ECG) analysis model using limited ECG data. We pretrained Vision Transformer (ViT) models by reconstructing the masked ECG data with MAE. We fine-tuned this MAE-based ECG pretrained model on ECG-echocardiography data from The University of Tokyo Hospital (UTokyo) for the detection of left ventricular systolic dysfunction (LVSD), and then evaluated it using multi-center external validation data from seven institutions, employing the area under the receiver operating characteristic curve (AUROC) for assessment. We included 38,245 ECG-echocardiography pairs from UTokyo and 229,439 pairs from all institutions. The performances of MAE-based ECG models pretrained using ECG data from UTokyo were significantly higher than that of other Deep Neural Network models across all external validation cohorts (AUROC, 0.913–0.962 for LVSD, *p* < 0.001). Moreover, we also found improvements for the MAE-based ECG analysis model depending on the model capacity and the amount of training data. Additionally, the MAE-based ECG analysis model maintained high performance even on the ECG benchmark dataset (PTB-XL). Our proposed method developed high performance MAE-based ECG analysis models using limited ECG data.

## Introduction

The generalization of deep neural network (DNN) algorithms to a broader population is an important challenge, and this problem can be a major barrier to the social implementation of DNN algorithms in the medical field [1, 2]. One of the most promising areas for the clinical implication of DNN algorithms has been 12-lead electrocardiogram (ECG) analysis. In previous studies about ECG, various analysis methods, including wavelet transformation and local binary patterns, have been utilized in ECG information processing [3–5]. Techniques such as Support Vector Machines, k-Nearest Neighbors, and particularly DNNs, including Convolutional Neural Networks (CNN), have been used for ECG wave form abnormality detection and arrhythmia detection, with DNNs demonstrating state-of-the-art performance [6–9]. Building on this foundation, researchers have achieved highly accurate detection of cardiac diseases from ECGs, tackling conditions that were previously challenging to diagnose [10, 11]. A typical example is left ventricular systolic dysfunction (LVSD), which is a common disease that significantly increases the risk of sudden death [12, 13]. Traditionally, the presence of LVSD is confirmed using echocardiographic findings in clinical practice. However, ECGs may offer a simpler screening method that can effectively bridge patients to the necessary echocardiographic examinations. Even in this cutting-edge field of ECGs, generalization performance is an essential issue in the implementation of DNN algorithms in clinical practice [2].

High-capacity models, such as Vision Transformers (ViTs), have attracted attention because they have been reported to improve generalizability by training using large amounts of data [14]. Generally, ViT comes in three model sizes: ViT-Base, ViT-Large, and ViT-Huge. Each has a different number of parameters: ViT-Base with about 86 million parameters, ViT-Large with around 300 million parameters, and ViT-Huge with approximately 600 million parameters. Models with larger capacities show higher performance [14]. However, training large capacity models requires large datasets. Obtaining such large amounts of data is often difficult because of the labeling costs of specialized knowledge and ethical considerations in the medical field.

To address this problem, self-supervised learning, which can make more effective use of limited data, has been considered in the field of computer vision tasks. Self-supervised learning using masked autoencoders (MAEs; licensed under an Attribution Non-Commercial 4.0 International License) has been applied to pretrain data-hungry models such as ViT-Base/Large/Huge [15] so that they can be trained on ImageNet [16] to improve generalization performance. Contrast-learning approaches, such as MoCo [17] and SimCLR [18], have also achieved high performance in computer vision tasks, but require image transformation to create a contrast with the original image. When using specialized medical images, medical expertise is essential to ensure that information does not lose its original meaning. By contrast, MAE learns a reusable representation of the input image by masking random patches and reconstructing missing pixels. Therefore, the transformations necessary in contrast learning are not required. Kim et al. in their 2023 study developed a Transformer-based deep learning model for atrial fibrillation and flutter segmentation in single-lead electrocardiograms using self-supervised learning with masked signal modeling. This model demonstrated excellent performance using both PhysioNet open-source databases and external validation data [19]. In another study utilizing data from the Apple Heart and Movement Study, self-supervised learning was employed to train foundation models for photoplethysmography and electrocardiogram recorded on Apple Watch. This research used data from thousands of participants and showed superior generalization capabilities for both photoplethysmography and electrocardiogram modalities, contributing to the development of new digital biomarkers [20]. Furthermore, a novel self-supervised learning approach that considers the spatio-temporal relationships inherent in electrocardiogram signals, called Spatio-Temporal Masked Electrocardiogram Modeling, was proposed. Spatio-Temporal Masked Electrocardiogram Modeling reconstructs masked areas of 12-lead electrocardiogram data to learn spatio-temporal features, outperforming other self-supervised learning baseline methods in arrhythmia classification tasks and demonstrating adaptability to various lead combinations [21]. These studies indicate that high-performance ECG analysis models can be developed using self-supervised learning, even when labeled data are limited. While there have been several studies utilizing transformers for ECG analysis, the exploration of MAE-based pretraining for a high-capability ViT dedicated to ECG analysis has been limited [19, 22].

In the present study, we aimed to develop an ECG analysis model with high performance for external validation cohort using ECG limited data. To achieve this, we tested the hypothesis that high-capacity ViT pretrained with MAE improves performance in downstream tasks compared with existing methods, and demonstrated the effective use of limited ECG data and the potential of high-capacity models in the medical field.

## Material and methods

### Data source and study population

We included patients aged ≥18 years who underwent echocardiography from January 2015 to May 2021 at eight academic medical institutions (UTokyo, The University of Tokyo Hospital; Mitsui, Mitsui Memorial Hospital; Asahi, Asahi General Hospital; Sakakibara, Sakakibara Heart Institute; Jichi, Jichi Medical University Saitama Medical Center; TokyoBay, Tokyo Bay Urayasu Ichikawa Medical Center; JR, JR Tokyo General Hospital; and NTT, NTT Medical Center Tokyo) and had a 12-lead ECG performed within 28 days of their echocardiography. We paired the echocardiography findings and 12-lead ECG (one-to-one) based on the respective examination dates. Echocardiography findings were only used for LVSD labeling on 12-lead ECGs, and echocardiography images were not analysed in this study. We used raw time series data from individual ECG leads (12-lead ECG of FUKUDA DENSHI, Tokyo, Japan or NIHON KOHDEN, Tokyo, Japan) as a 10-second interval with a sampling rate of 500 Hz. When a single echocardiography examination corresponded to multiple ECG examinations, we selected the closest ECG examination to that echocardiography examination.

### Definition of LVSD

LVSD was defined as an ejection fraction of less than 40%. All echocardiography examinations had been performed by skilled ultrasound sonographers or cardiologists, and all echocardiography findings had been interpreted by one or two experienced echocardiologists. ECG-echocardiography pairs that indicated LVSD were labelled 1, and the rest were labelled 0. We excluded ECG–echocardiography pairs that were missing data regarding the assessment of LVSD.

### ECG internal cohort and external validation cohorts

To build MAE-based ECG models, we defined the internal cohort as the echocardiography and 12-lead ECG dataset from UTokyo. We separated the internal cohort from UTokyo into training, validation, and test datasets with a 7:1.5:1.5 ratio according to a random number. Note that to avoid data leakage, we assigned all ECGs from one patient to the same split.

To evaluate the performance of the ECG models trained on UTokyo, the validation cohorts were defined as the echocardiography and 12-lead ECG dataset collected from seven institutions (Mitsui, Asahi, Sakakibara, Jichi, TokyoBay, JR, and NTT), and performed external validation. We excluded ECG–echocardiography pairs without data regarding the assessment of LVSD. For a rigorous evaluation of performance, we completely isolated the training datasets and external validation cohorts used in this study on a per-institution basis.

### Details of the internal cohort

**Fig 1:**
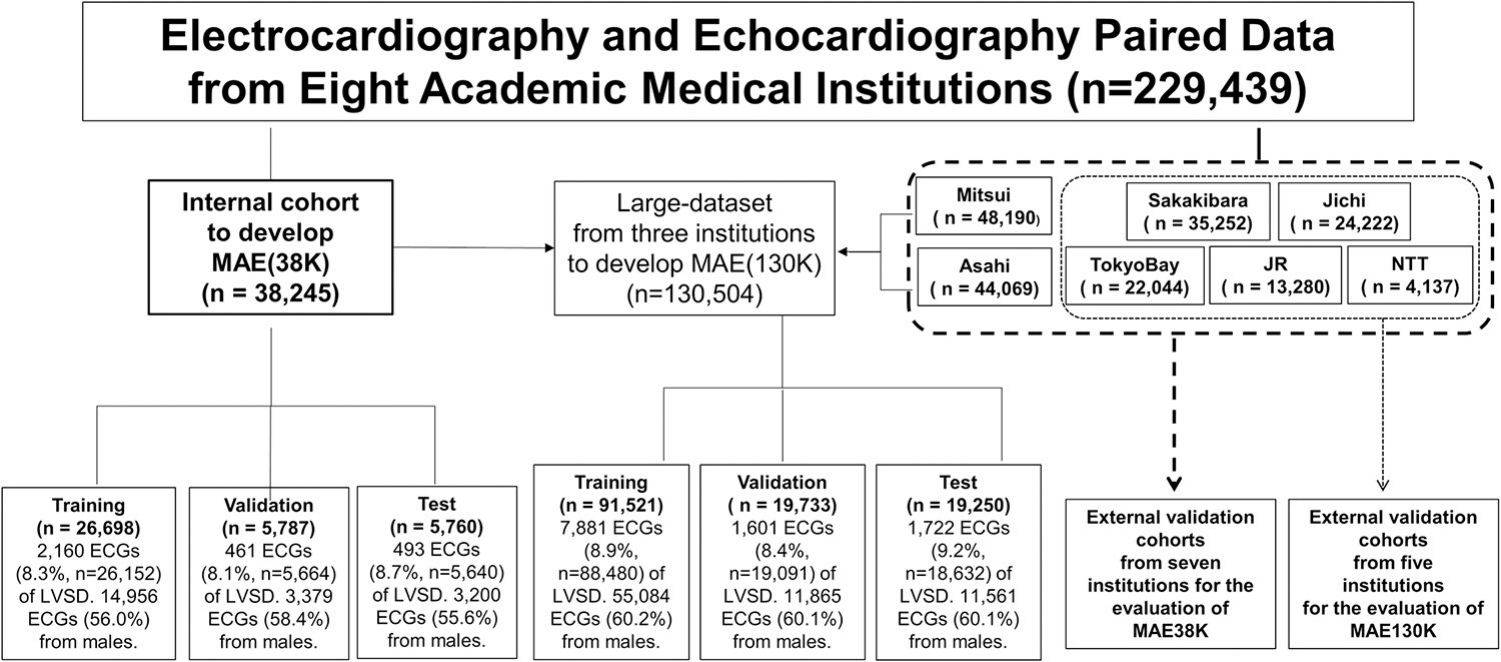
Flow chart for splitting the datasets. Flow chart showing how the datasets used for model training and validation were created. LVSD, left ventricular systolic dysfunction; MAE38K, Vision Transformers pretrained on ECG data from UTokyo using a masked autoencoder; Large-dataset, electrocardiography and echocardiography paired dataset from three institutions (UTokyo, Mitsui, and Asahi); MAE130K, Vision Transformers pretrained on ECG data from three institutions (UTokyo, Mitsui, and Asahi).

A total of 38,245 ECG–echocardiography record pairs from 23,358 patients were included, and 7,029 patients had multiple pairs. The baseline characteristics of the patients included in the internal cohort from UTokyo are shown in Table 1 and Fig 1. The mean age of the study population of UTokyo was 63.3±17.0 years. There were 21,535 12-lead ECGs (56.3%) from 12,788 male patients and 16,710 12-lead ECGs (43.7%) from 10,570 female patients. In the internal cohort, there were 3,114 ECGs (8.3%) from 1,400 patients with LVSD. The training, validation, and internal test datasets included 26,698 12-lead ECGs (69.8%) from 16,321 patients, 5,787 12-lead ECGs (15.1%) from 3,513 patients, and 5,760 12-lead ECGs (15.1%) from 3,524 patients. More details of the patient characteristics of the internal cohort are shown in Table 2.

**Table 1:** Study-level demographic information.

Variables	UTokyo	Mitsui	Asahi	Sakakibara	Jichi	TokyoBay	JR	NTT
Number of studies	38,245	48,190	44,069	35,252	24,222	22,044	13,280	4,137
Number of patients	23,358	23,682	23,940	19,442	15,829	13,607	8,116	3,611
Age, years ±SD	63.3±17.0	69.9±13.9	70.1±13.4	68.2±16.2	69.0±13.7	69.4±15.0	67.1±15.5	N/A
Age, groups								
≤30	2,189(5.7)	459(1.0)	593(1.3)	1271(3.6)	426(1.8)	385(1.7)	338(2.5)	N/A
30–50	6,475(16.9)	4,637(9.6)	3,294(7.5)	3,861(11.0)	2,268(9.4)	2,453(11.1)	1,614(12.2)	N/A
50–70	13,778(36.0)	16,864(35.0)	16,423(37.3)	11,366(32.2)	8,322(34.4)	7,112(32.3)	5,119(38.5)	N/A
70–90	15,486(40.5)	24,837(51.5)	22,664(51.4)	17,904(50.8)	12,894(53.2)	11,373(51.6)	5,740(43.2)	N/A
>90	317(1.0)	1,393(2.9)	1,095(2.5)	850(2.4)	312(1.3)	721(3.3)	469(3.5)	N/A
Sex		n = 48,186						
Female, n (%)	16,710(43.7)	17,863(37.1)	17,417(39.5)	15,123(42.9)	9,133(37.7)	9,411(42.7)	4,803(36.2)	1,511(36.5)
Male, n (%)	21,535(56.3)	30,323(62.9)	26,652(60.5)	20,129(57.1)	15,089(62.3)	12,633(57.3)	8,477(63.8)	2,626(63.5)
Body height, cm ±SD	161.9±16.8(n = 37,379)	161.7±14.0 (n = 46,771)	159.7±16.2 (n = 33,537)	161.0±10.2 (n = 34,891)	161.1±9.7 (n = 21,021)	N/A	162.3±10.1 (n = 13,044)	N/A
Body weight, kg ±SD	60.4±14.3(n = 37,384)	61.1±14.8 (n = 46,909)	60.7±14.3 (n = 34,500)	60.1±13.4 (n = 34,893)	60.6±13.4 (n = 21,760)	N/A	62.1±14.6 (n = 13,041)	N/A
Mean EF, (%) ±SD	61.6±14.2 (n = 37,246)	61.1±13.2 (n = 47,353)	59.7±13.5 (n = 41,394)	56.8±10.5 (n = 34,841)	59.3±14.3 (n = 24,205)	55.3±11.8 (n = 21,676)	65.1±12.0 (n = 12,759)	64.0±11.6 (n = 4,124)
LVSD, n(%)	3,114 (n = 37,456, 8.3)	4,187 (n = 47,353, 8.8)	3,903 (n = 41,394, 9.4)	3,181 (n = 34,841, 9.1)	2,947 (n = 24,205, 12.2)	2,724 (n = 21,676, 12.6)	595 (n = 12,759, 4.7)	199 (n = 4,124, 4.8)
Manufacturer	FUKUDA DENSHI	FUKUDA DENSHI	NIHON KOHDEN	NIHON KOHDEN	NIHON KOHDEN	NIHON KOHDEN	NIHON KOHDEN	NIHON KOHDEN

Data are expressed as mean ± standard deviation or number (percentage).

EF, ejection fraction; and LVSD, left ventricular systolic dysfunction.

**Table 2:** Patient characteristics in the internal cohort.

Variables	Training dataset (n = 26,698, 69.8%)	Validation dataset (n = 5,787, 15.1%)	Test dataset (n = 5,760, 15.1%)	*P*-value
Participant characteristics				
Age, (years) *	63.2±17.0	63.4±17.1	63.4±17.2	0.563
Age, groups				
≤30	1,496(5.6)	338(5.8)	355(6.2)	0.107
30–50	4,602(17.2)	933(16.1)	940(16.3)	
50–70	9,677(36.2)	2,079(35.9)	2,022(35.1)	
70–90	10,704(40.1)	2,387(41.2)	2,395(41.6)	
>90	219(0.8)	50(0.9)	48(0.8)	
Sex				
Female, n (%)	11,742(44.0)	2,408(41.6)	2,560(44.4)	0.002
male, n (%)	14,956(56.0)	3,379(58.4)	3,200(55.6)	
Body height, cm ±SD *	161.9±16.6 (n = 26,094)	162.4±22.2 (n = 5,664)	161.6±9.7 (n = 5,618)	0.053
Body weight, kg ±SD *	60.4±14.1 (n = 26,097)	60.8±13.8 (n = 5,664)	60.0±15.4 (n = 5,623)	0.020
Mean EF, (%) ±SD *	61.6±14.1 (n = 26,152)	61.6±14.2 (n = 5,664)	61.6±14.7 (n = 5,640)	0.990
LVSD, n(%)	2,160 (n = 26,152, 8.3)	461 (n = 5,664, 8.1)	493 (n = 5,640, 8.7)	0.431

Data are expressed as mean ± standard deviation or number (percentage).

Pearson’s chi-square test was used for categorical variables. Student’s *t*-test was used for normally distributed continuous variables, and the Mann–Whitney U test was used for non-normally distributed continuous variables. For non-normally distributed continuous variables, an asterisk (*) have been marked next to each variable’s name.

EF, ejection fraction; and LVSD, left ventricular systolic dysfunction.

### Details of the external validation cohorts

From the seven participating institutions, there were 48,190; 44,069; 35,252; 24,222; 22,044; 13,280; and 4,137 eligible ECG–echocardiography pairs from 23,682; 23,940; 19,442; 15,829; 13,607; 8,116; and 3,611 patients at Mitsui, Asahi, Sakakibara, Jichi, TokyoBay, JR, and NTT, respectively (Table 1 and Fig 1). The results for the prevalence of LVSD were 8.8%, 9.4%, 9.1%, 12.2%, 12.6%, 4.7%, and 4.8% for Mitsui, Asahi, Sakakibara, Jichi, TokyoBay, JR, and NTT, respectively. Of the patients at the seven institutions, 62.9%, 60.5%, 57.1%, 62.3%, 57.3%, 63.8%, and 63.5% were male. the mean ages were 69.9±13.9 years, 70.1±13.4 years, 68.2±16.2 years, 69.0±13.7 years, 69.4±15.0years, and 67.1±15.5 years for Mitsui, Asahi, Sakakibara, Jichi, TokyoBay, and JR, respectively, and there were no data on age from NTT. More details of the patient characteristics of each cohort are shown in Table 1.

### ECG model pretraining with MAE

In the first step of our approach, we pretrained the ViT- Base/Large/Huge encoders using MAE-based self-supervised learning on all 38,245 ECG data for the internal cohort from UTokyo. We use ViT- architecture to take advantage of its strong generalization capabilities. Each ViT encoder trained on the internal cohort is represented as ViT- Base38K/Large38K/Huge38K. In an original MAE for RGB image data (e.g., 3×224×224), the input was divided into 16 × 16 patches. These patches were randomly masked and then reconstructed the missing pixels. This process created an encoder that had learned a useful representation [15].

**Fig 2:**
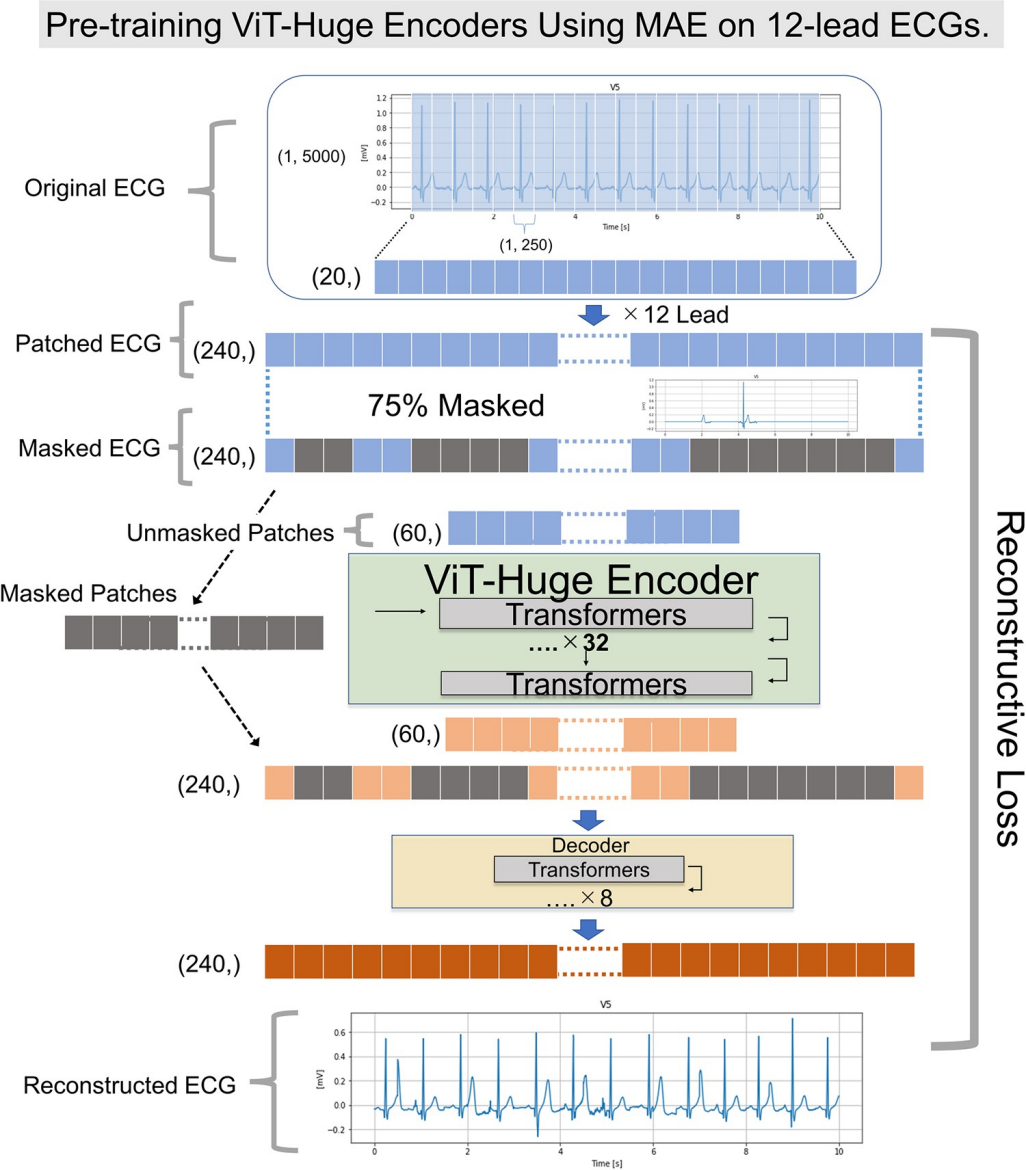
Network architecture of MAE for 12-lead ECGs. This figure shows the network architecture of MAE-based self-supervised learning for 12-lead ECGs. The ViT-Huge is used as an example in the figure. We treated original ECG data from each lead as a 1×5000 matrix of the ECG voltage. The input ECG data was divided into 1 × 250 patches and voltage data from each lead is converted into 20 patch sequences, which is 240 patch sequences for 12-lead ECGs. These patches were randomly masked and only unmasked patches (60 patch sequences) were input to the MAE encoder. We used ViT-Huge encoder for the MAE encoder. ViT-Huge encoder then output 60 encoded patch sequence with 1280-dimensional feature vectors. For the input to the MAE decoder, the full set of patches consisting of encoded patches and masked patches were applied. Proposing MAE reconstructs the input by predicting the voltage values for each masked patch of 12-lead ECGs. Each element in the decoder’s output is a vector of voltage values representing a patch. The last layer of the decoder is a linear projection whose number of output channels equals the number of inputs. Loss function computes the mean squared error as reconstructive loss between the reconstructed and original 12-lead ECGs. Same as original MAE, we compute the loss only on masked patches. These processes could create ViT-Huge encoders for 12-lead ECGs with high performances for downstream task. Other implementation details followed those in a previous study [15]. In this study, while we use ViT-Large and ViT-Base as well, the primary model employed is ViT-Huge. Given that the structure of MAE does not change with the size of the ViT model, Fig 2 is presented using ViT-Huge as an example. MAE, Masked Autoencoder; ECG, electrocardiography; ViT, Vision transformer.

**Fig 3:**
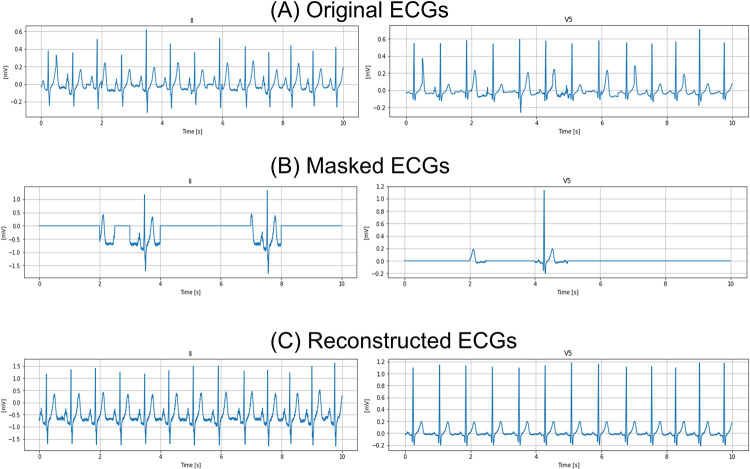
Example of the reconstruction process in II and V5 lead. (A) Original ECGs; (B) masked ECGs; and (C) reconstructed ECGs.

On the other hand, raw data from each ECG record were represented as a 12×5000 matrix of the ECG voltage, in which the first dimension was a spatial dimension (each column represented one lead) and the second dimension was the temporal dimension (each row represented a specific time point). In an effort to minimize alterations to the original MAE’s architecture, we added a single-channel dimension to the 12×5000 ECG data, processing the ECGs in a shape of 1×12×5000. To take advantage of the interrelationship between the 12 ECG leads, we changed the patch size to 1×250. As a result, the ECG information per patch was 0.5 sec. In self-supervised pretraining, we input ECGs after normalization into the encoder using MAE. We did not utilize additional data preprocessing methods such as band-pass filtering, removing baseline wandering, or noise removal in our analysis. For self-supervised learning, we randomly masked 75% of the ECG time series and pretrained ViT encoders by reconstructing the masked part with MAE. We trained the ViT- Base/Large/Huge encoders for 1600 epochs and the batch sizes for ViT-Base/Large/Huge are 1536/1280/768, which is the largest batch size in our development environment. We trained models without Automatic Mixed Precision using gradient clipping to stabilize the pretraining process. The detail network architecture of MAE for 12-lead ECGs is shown in Fig 2. Other implementation details followed those in a previous study [15]. We used four sets of Nvidia Tesla A100 80 GB graphics processing units (Nvidia Corporation, Santa Clara, USA). To obtain a qualitative sense of our reconstruction task (see Fig 3 and S1 Fig in S1 File). These figures show an example sampled from an internal cohort consisting of UTokyo data using ViT-Large 38K.

### Performance evaluation on downstream task

We fine-tuned the MAE-based ECG model on the downstream task. In this study, the downstream task was the detection of LVSD using 12-lead ECGs. LVSD is one of the most important echocardiographic findings that can be detected from 12-lead ECGs. As a baseline model, we used a two-dimensional convolutional neural network (Baseline-CNN) because, in a previous study, researchers showed that this architecture achieved high performance in detecting LVSD [10]. Baseline-CNN consisted of six temporal convolution blocks, one spatial convolution block, and one fully connected layer. Baseline-CNN used a 12×5000 ECG matrix as input, and finally output a 128-dimensional feature vector. We fine-tuned the Baseline-CNN model with randomly initialized model weights. Additionally, we also fine-tuned a ViT encoder with the model weights pretrained by the MAE using ImageNet-1K (ViT-IN1K, https://dl.fbaipublicfiles.com/mae/pretrain/mae_pretrain_vit_large.pth [15]) as a subject of comparison for the downstream task. In the downstream task, we added two fully connected layers and a sigmoid layer. For detail comprehensive evaluation, we also provided the Resnet1d and Inceptiontime. The Resnet1d (https://github.com/hsd1503/resnet1d.git) is the CNN, one-dimensional adaptations of the popular Resnet-architecture [23] from computer vision, which showed very good perform in several ECG classification studies [24, 25]. Inceptiontime (https://github.com/hfawaz/InceptionTime.git [26]) is the neural network architecture including the inception module, which is known for its excellent performance in time series data analyzing [26].

The ECG data obtained from the training dataset were inputted into the models. All models were trained to minimize binary cross-entropy loss between the models’ predictions and ground truth labels using the Adam optimizer. The batch size was chosen from among 32, 64, 128, and 256, and the initial learning rate was chosen from among 0.001, 0.0001, 0.00001, and 0.000001. The training was conducted for 100 epochs, and if the loss did not decrease for 3 epochs, the learning rate was reduced by a factor of 1/2. If the loss did not decrease for 10 consecutive epochs, model training was stopped, and the model’s weights at the lowest loss value were saved, even if 100 epochs had not been completed. In the analysis, we did not use data augmentation or transformations to demonstrate pure model performance. We compared the performances of the created models to detect LVSD using the area under the receiver operating characteristic curve (AUROC) for the test dataset and the external validation cohorts from seven institutions. AUROC represents the area under the receiver operating characteristic curve (ROC) [27]. ROC is a summary measure that examines the performance of a model at various cutoffs, plotting the True Positive Rate (Recall) on the y-axis and the False Positive Rate (1-Specificity) on the x-axis [27]. The value of AUROC ranges from 0 to 1, with higher values indicating better model performance [27]. An AUROC above 0.9 indicates high performance, between 0.7 and 0.9 indicates moderate performance, between 0.5 and 0.7 indicates low performance, and 0.5 indicates random chance [27].

### Training and evaluation on a large dataset

To confirm the dependence of MAE-based ECG model performance on the amount of data pretrained using our proposed method, we pretrained the ViT-Huge encoder on a larger ECG dataset with MAE and evaluated its performance in the downstream task. We created a Large-dataset using all 130,504 ECG data from three data-rich institutions (UTokyo, Mitsui, and Asahi). All the ECG data from the Large-dataset was used to pretrain the ViT-Huge encoder (ViT-Huge130K). For fine-tuning, we separated the Large-dataset from three institutions into training, validation, and internal test datasets with a 7:1.5:1.5 ratio according to a random number. Note that to avoid data leakage, as mentioned above, we assigned all ECGs from one patient to the same split. Detailed patient characteristics for the Large-dataset are shown in S1 Table in S1 File. We evaluated the performances of the created models using the AUROC for the internal test dataset from the large-dataset and the external validation cohorts from five institutions that were not used for training.

### Investigating the impact of masking ratios and masking strategies for MAE

To investigate the impact of masking ratios on the performance of MAE-based ECG models, we conducted experiments with masking rates of 50%, 75%, and 90% for MAE-based self-supervised learning with random masking. Moreover, to assess the effectiveness of different masking strategies, we introduced a pretraining model utilizing random masking, grid-based masking, and random per lead masking for each lead. In the examination of different masking strategies, the masking ratio is set at 75%. In these analyses, we evaluated the performance of the ViT-Large 38K model to detect LVSD using the AUROC for the test dataset in the Internal Cohort, which consists of UTokyo data. For a better understanding of masking strategy, please refer to S1-S3 Figs in S1 File.

### Performance evaluation on the benchmark dataset

Because the ECG data used in this study were collected at an academic medical centre in Japan and did not include data from different countries, we evaluated the performance of the MAE-based ECG-pretrained model on the benchmark dataset PTB-XL [24]. PTB-XL is 12-lead ECG dataset that comprises 21,837 records from 18,885 patients of 10-second length at 500 Hz and a downsampled version at 100 Hz. This dataset consists of 71 labels, and the evaluation task is framed as a multi-label classification task. It is worth noting that these labels cover a wide variety of diagnostic, form, and rhythm statements and can be used to perform a comprehensive evaluation of ECG analysis algorithms. The 44 diagnostic statements can be categorized in terms of five super-classes (normal/conduction disturbance/myocardial infarction/hypertrophy/ST-T change); the 19 form statements are relevant to mostly morphological changes in specific ECG segments, such as an abnormal QRS complex; and the 12 rhythm statements comprise statements that characterize normal cardiac rhythms, in addition to arrhythmia [24, 28]. The dataset is organized into ten stratified, label-balanced folds, where the first eight are used as the training set, the ninth is used as the validation set, and the tenth is used as the test set [24]. The dataset summaries of the PTB-XL dataset refer to previous studies [24, 28]. We fine-tuned ViT-Huge130K for multi-label classification tasks for all statements (71 classifications), diagnostic statements (44 classifications), form statements (19 classifications), and rhythm statements (12 classifications) using 10-second length 12-lead ECG data sampled at 500 Hz.

### Statistical analysis

We presented continuous variables as the mean and standard deviation, and compared them using two-tailed analysis of variance. Categorical variables were expressed as the frequency and percentage, and compared them using chi-square tests. For the LVSD detection task, models were created using the holdout method, and the model performances were verified with external validation data to confirm the performance. The model performances were evaluated using the AUC and 95% confidence interval. For the analysis of PTB-XL, we evaluated the model performances based on averaging class-wise AUROCs over all classes (macro-AUC) and compared this with benchmark scores [24, 29]. As in previous studies, we reported the mean and standard deviation of macro-AUC over ten fine-tuning runs [24, 28]. For example, we described a macro-AUC of 0.960 with a standard deviation of 0.002 as 0.960 (02). We used the DeLong method to compare AUROC values [30]. We performed statistical analysis using JMP Pro 16 (SAS Institute, Tokyo, Japan), and defined statistical significance as a *p*-value < 0.05.

### Ethics statement

The study was conducted in accordance with the revised Declaration of Helsinki and approved by the institutional review boards of The University of Tokyo Hospital (2021132NI-(1)). The participating institutions were also collectively reviewed by the review boards of The University of Tokyo Hospital. Informed consent was obtained in the form of opt-outs on websites.

## Results

### MAE improved the performance of the LVSD detection model

**Fig 4:**
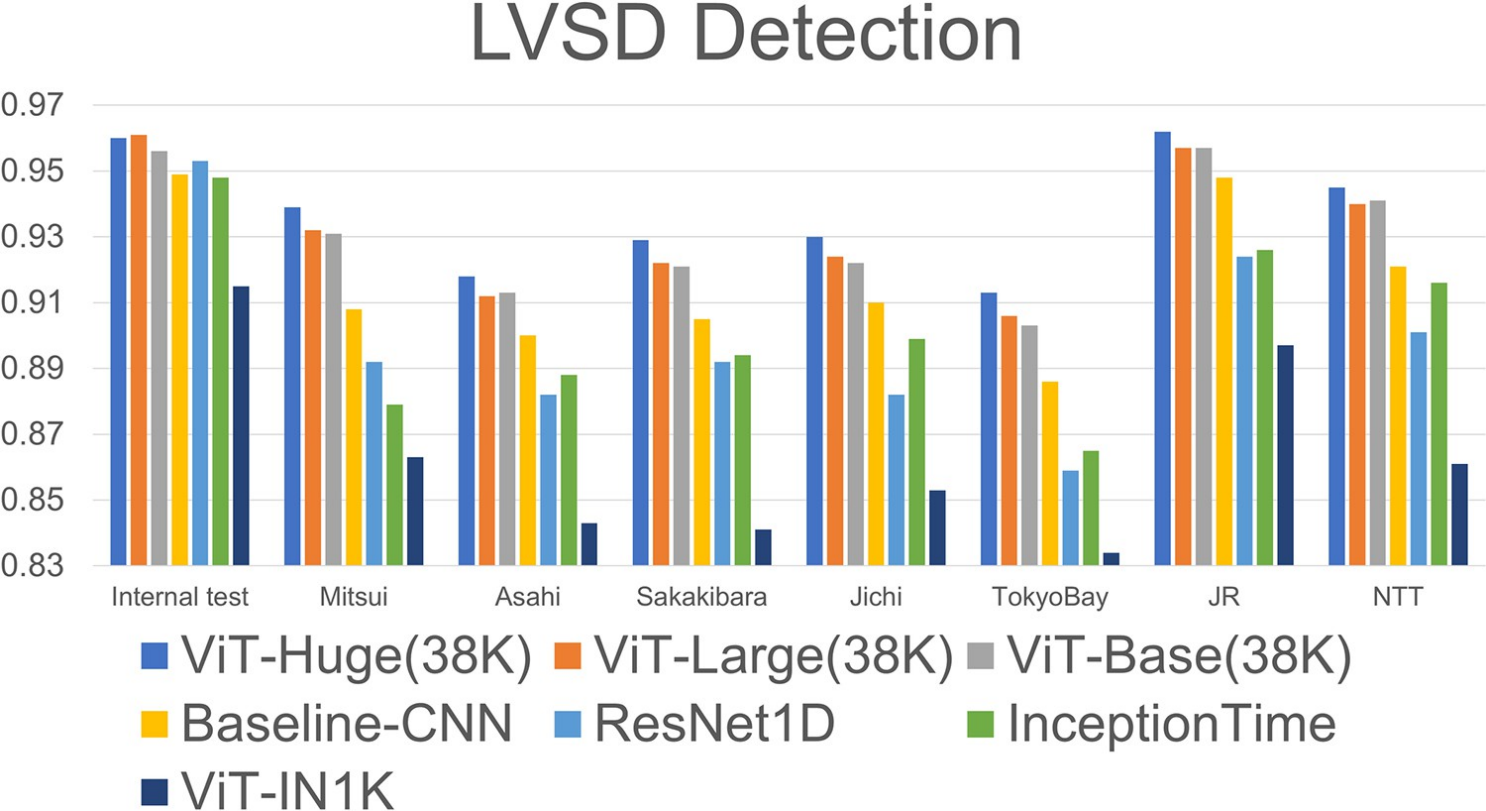
Model performance values used to detect LVSD from 12-lead ECGs on the internal test dataset and external validation cohorts. The bars indicate the AUROC for LVSD detection of models on the internal test dataset and validation cohorts of Mitsui, Asahi, Sakakibara, Jichi, TokyoBay, JR, and NTT. LVSD, left ventricular systolic dysfunction; AUROC, area under the receiver operating characteristics curve; ViT-Huge38K, Vision Transformer Huge pretrained on ECG data from UTokyo using a masked autoencoder; ViT-Large38K, Vision Transformer Large pretrained on ECG data from UTokyo using a masked autoencoder; ViT-Base38K, Vision Transformer Base pretrained on ECG data from UTokyo using a masked autoencoder; Baseline-CNN, two-dimensional convolutional neural network; ViT-IN1K, Vision Transformer pretrained on ImageNet-1K using a masked autoencoder.

For the internal test dataset of UTokyo, the AUROC of the pretrained ViT-Huge38K was 0.960 (95% confidence interval (CI): 0.950–0.970), which was significantly greater than that of the Baseline-CNN model (0.949; 95% CI: 0.938–0.959; *p* = 0.001) and baseline ViT-IN1K (0.915; 95% CI: 0.900–0.928; *p* < 0.001). ViT-Huge38K detected LVSD with high performance, with significant differences for the external validation cohorts (AUROC, 0.939, 0.918, 0.929, 0.930, 0.913, 0.962, and 0.945 for Mitsui, Asahi, Sakakibara, Jichi, TokyoBay, JR, and NTT, respectively) compared with the Baseline-CNN (AUROC, 0.908, 0.900, 0.905, 0.910, 0.886, 0.948, and 0.921 for Mitsui, Asahi, Sakakibara, Jichi, TokyoBay, JR, and NTT; *p* < 0.001, respectively) and baseline ViT-IN1K (AUROC, 0.863, 0.843, 0.841, 0.853, 0.834, 0.897, and 0.861 for Mitsui, Asahi, Sakakibara, Jichi, TokyoBay, JR, and NTT; *p* < 0.001, respectively). Detailed model performance values including Resnet1D and Inceptiontime used to detect LVSD for the internal test dataset and external validation cohorts are shown in Fig 4 and Table 3.

**Table 3:** Model performances for LVSD detection.

	Test dataset of Internal Cohort	Test dataset of Large-dataset	Mitsui	Asahi	Sakakibara	Jichi	TokyoBay	JR	NTT
Model	AUROC	AUROC	AUROC	AUROC	AUROC	AUROC	AUROC	AUROC	AUROC
ViT- Huge38K	0.960	0.942	0.939	0.918	0.929	0.930	0.913	0.962	0.945
	(95% CI: 0.950–0.970)	(95% CI: 0.936–0.947)	(95% CI: 0.935–0.942)	(95% CI: 0.914–0.922)	(95% CI: 0.924–0.933)	(95% CI: 0.926–0.934)	(95% CI: 0.907–0.918)	(95% CI: 0.956–0.967)	(95% CI: 0.926–0.959)
ViT-Large38K	0.961	-	0.932	0.912	0.922	0.924	0.906	0.957	0.940
	(95% CI: 0.950–0.969)	-	(95% CI: 0.928–0.935)	(95% CI: 0.908–0.917)	(95% CI: 0.918–0.926)	(95% CI: 0.919–0.928)	(95% CI: 0.900–0.911)	(95% CI: 0.951–0.963)	(95% CI: 0.922–0.954)
ViT-Base38K	0.956	-	0.931	0.913	0.921	0.922	0.903	0.957	0.941
	(95% CI: 0.946–0.963)	-	(95% CI: 0.927–0.934)	(95% CI: 0.909–0.918)	(95% CI: 0.917–0.925)	(95% CI: 0.917–0.926)	(95% CI: 0.898–0.909)	(95% CI: 0.950–0.963)	(95% CI: 0.921–0.956)
ViT-Huge130K	-	0.953	-	-	0.942	0.938	0.928	0.967	0.952
	-	(95% CI: 0.948–0.957)	-	-	(95% CI: 0.939–0.946)	(95% CI: 0.934–0.942)	(95% CI: 0.923–0.932)	(95% CI: 0.962–0.971)	(95% CI: 0.934–0.966)
Base-line CNN	0.949	-	0.908	0.900	0.905	0.910	0.886	0.948	0.921
	(95% CI: 0.938–0.959)	-	(95% CI: 0.904–0.912)	(95% CI: 0.895–0.904)	(95% CI: 0.901–0.910)	(95% CI: 0.906–0.915)	(95% CI: 0.880–0.892)	(95% CI: 0.940–0.954)	(95% CI: 0.898–0.939)
Resnet1D	0.953	-	0.892	0.882	0.892	0.882	0.859	0.924	0.901
	(95% CI: 0.943–0.961)	-	(95% CI: 0.887–0.896)	(95% CI: 0.876–0.887)	(95% CI: 0.887–0.898)	(95% CI: 0.876–0.888)	(95% CI: 0.852–0.866)	(95% CI: 0.914–0.934)	(95% CI: 0.876–0.921)
Inceptiontime	0.948	-	0.879	0.888	0.894	0.899	0.865	0.926	0.916
	(95% CI: 0.938–0.957)	-	(95% CI: 0.873–0.884)	(95% CI: 0.883–0.894)	(95% CI: 0.888–0.899)	(95% CI: 0.893–0.904)	(95% CI: 0.858–0.872)	(95% CI: 0.915–0.936)	(95% CI: 0.891–0.935)
ViT-IN1K	0.915	-	0.863	0.843	0.841	0.853	0.834	0.897	0.861
	(95% CI: 0.900–0.928)	-	(95% CI: 0.837–0.849)	(95% CI: 0.834–0.849)	(95% CI: 0.835–0.847)	(95% CI: 0.847–0.859)	(95% CI: 0.827–0.841)	(95% CI: 0.887–0.908)	(95% CI: 0.836–0.883)

LVSD, left ventricular systolic dysfunction; Internal cohort, the echocardiography and 12-lead ECG dataset from UTokyo. Large-dataset, electrocardiography and echocardiography paired dataset from three institutions (UTokyo, Mitsui, and Asahi); ViT-Huge38K, Vision Transformer Huge pretrained on ECG data from UTokyo using a masked autoencoder; CI, confidence interval; ViT-Large38K, Vision Transformer Large pretrained on ECG data from UTokyo using a masked autoencoder; ViT-Base38K, Vision Transformer Base pretrained on ECG data from UTokyo using a masked autoencoder; ViT-Huge130K, Vision Transformer-Huge pretrained on ECG data from three institutions (UTokyo, Mitsui, Asahi); Baseline-CNN, two-dimensional convolutional neural network; ViT-IN1K, Vision Transformer pretrained on ImageNet-1K using a masked autoencoder.

### The performance of pretrained model increases with capacity

To demonstrate that the performance of MAE-based ECG models improves with increasing capacity: base, large, and huge, we compared the performances of these three models. ViT-Huge38K showed high performance for external validation cohort compared to the performance of ViT-Large38K (AUROC of LVSD detection model, 0.932, 0.912, 0.922, 0.924, 0.906, 0.957, 0.940 for the Mitsui, Asahi, Sakakibara, Jichi, TokyoBay, JR, and NTT external validation cohorts; *p* < 0.001, *p* < 0.001, *p* < 0.001, *p* < 0.001, *p* < 0.001, *p* = 0.003, and *p* = 0.087, respectively) and ViT-Base38K (AUROC of LVSD detection model, 0.931, 0.913, 0.921, 0.922, 0.903, 0.957, 0.941 for the Mitsui, Asahi, Sakakibara, Jichi, TokyoBay, JR, and NTT external validation cohorts; *p* < 0.001, *p* < 0.001, *p* < 0.001, *p* < 0.001, *p* < 0.001, *p* = 0.007, and *p* = 0.084, respectively). On the other hand, in the internal test data, there was no significant difference in AUROC for LVSD detection due to ViT’s model capacities (AUROC of LVSD detection model, ViT-Huge38K 0.960, ViT-Large38K 0.961, ViT-Base38K 0.956 for internal test; *p* = 0.886 and 0.056, respectively, compared to ViT-Huge38K).

### A large amount of data improved the model performance

The MAE-pretrained and fine-tuned model on ECG data from three institutions enhanced the overall discrimination of the ECG model and greatly improved the performance to five external cohorts. The AUROCs of the model used to detect LSVD were 0.953 for the internal test on the Large-dataset, and 0.942, 0.938, 0.928, 0.967, and 0.952 for the Sakakibara, Jichi, TokyoBay, JR, and NTT external validation cohort, respectively, which were significantly greater than those of ViT-Huge38K trained on ECG data from a single institution (AUROC of the LVSD detection model, 0.942 for the internal test on the Large-dataset; *p* = 0.001; and 0.929, 0.930, 0.913, and 0.962 for the Sakakibara, Jichi, TokyoBay, and JR external validation cohorts; *p* < 0.001, *p* < 0.001, *p* < 0.001, and *p* = 0.004, respectively) as shown in Table 3. Although this analysis did not find a significant difference for NTT, the performances of the models were sufficiently compatible.

### A high masking ratio and random masking strategy enhance the performance

We examined the effect of masking ratios of 50%, 75%, and 90% with randomly chosen masked regions. The results showed that the model achieved the highest AUROC of 0.961 (95% CI: 0.950–0.970) at a 75% masking ratio, followed closely by a 90% ratio with an AUROC of 0.959 (95% CI: 0.947–0.968), and a 50% ratio yielding an AUROC of 0.948 (95% CI: 0.937–0.958). And then, we explored three masking strategies—random, grid-based, and random per lead at a fixed 75% masking ratio. The random masking strategy led to the highest AUROC of 0.961 (95% CI: 0.950–0.970), outperforming grid-based masking with an AUROC of 0.949 (95% CI: 0.940–0.958) and random per lead masking, which achieved an AUROC of 0.955 (95% CI: 0.944–0.963). These findings indicate that both higher masking ratios and the random masking strategy enhance the performance of MAE-based ECG models (S2, S3 Tables in S1 File).

### MAE-based ECG models achieved powerful performance on the benchmark dataset

For the benchmark dataset, the macro-AUCs of the MAE-based ViT-Huge 130K were 0.930 (04), 0.940 (02), 0.883 (01), and 0.970 (02) for all statements, diagnostic statements, form statements, and rhythm statements, respectively. ViT-Huge 130K outperformed the ViT-Huge 38K across all benchmark tasks. The ViT-Huge 130K performance for all statements, diagnostic statements, and rhythm statements exceeded that of the benchmark score, with the rhythm statement being the most notable, as shown in Table 4. The form statement, while not quite matching the benchmark, achieved competitive performance, as shown in Table 4.

**Table 4:** MAE-based ECG model performances on the benchmark dataset (*PTB-XL* test fold).

	ViT-Huge 38K	ViT-Huge130K	Benchmark score
Task	Macro-AUROC	Macro-AUROC	Macro-AUROC
All statements (71 classes)	0.919(03)	0.930(04)	0.925(08)
Diagnostic statement (44 classes)	0.938(03)	0.940(02)	0.937(08)
Form statement (19 classes)	0.833(01)	0.883(01)	0.899(22)
Rhythm statement (12 classes)	0.938(09)	0.970(02)	0.957(19)

Benchmark scores are based on reference (30).

MAE, Masked autoencoder; PTB-XL test fold, Test dataset that PTB-XL provides in advance for testing(24); ViT-Huge38K, Vision Transformers pretrained on ECG data from UTokyo using a masked autoencoder; ViT-Huge130K, Vision Transformer-Huge pretrained on ECG data from three institutions (The University of Tokyo Hospital, Mitsui Memorial Hospital, and Asahi General Hospital); AUROC indicates the area under the receiver operating characteristic curve. As before, we report the mean and standard deviation of Macro-AUROC over ten fine-tuning runs in the same manner as benchmark studies. For example, we described a macro-AUC of 0.960 with a standard deviation of 0.002 as 0.960 (02).

## Discussion

We developed an MAE-based ECG-pretrained model, and the model achieved high performance on external validation cohorts for downstream task. Model performance improved depending on the amount of data, outperforming that on a benchmark dataset. The MAE-based ECG-pretrained model could be useful for various ECG-based tasks.

The MAE developed a high-capacity ViT with excellent performance using limited ECG data. In this study, ViT-Huge demonstrated higher performance compared to ViT-Large and ViT-Base, indicating that the performance of pretrained ViT with MAE improves depending on its capacity. Generally, training large capacity models requires large datasets. However, obtaining large amounts of data is often difficult because of the labeling costs of specialized knowledge and ethical considerations in the medical field. There has been no previous study in which researchers have shown that a model as large as ViT-Huge can be effectively pretrained with a small number of ECG data. In this study, our proposed MAE succeeded in creating models with performance using limited ECG data.

To demonstrate the relationship between the amount of training data and model performance for MAE-based models, we compared the performance of an MAE-based model trained on the Large-dataset that consisted of data from three institutions with that of an MAE-based model trained on a dataset that consisted of data from a single institution (UTokyo). The ECG dataset from the three institutions contained 130,504 ECG data, whereas that from the single institution contained 38,245 ECG data. The results show that the model trained on the larger dataset performed much better on the internal test dataset and on the external validation cohorts from the five institutions. This result is consistent with previous studies in which researchers showed that ViTs achieved higher performance when trained on sufficiently large-scale data [14]. Our results suggest that it is possible to further improve model performance using larger data.

We discuss comparisons of model performances. First, ViT-IN1K which is not pre-trained with MAE clearly performed worse than MAE-based ECG model, based on ViT, because it is known that ViT requires a large amount of data for training compared to CNN due to the lack of induction bias. The amount of data used in this study was not sufficient to train ViT, which has not been pre-trained for ECG with MAE. On the other hand, The Baseline-CNN, Resnet1d, and Inceptiontime, based on CNN architecture, which are suited for learning with less amount of data due to the effect of induction bias, show high performance on internal test data. However, Resnet1d and Inceptiontime, which have performed well in previous studies, show lower performance on external validation data than Baseline-CNN of small architectures. Weaknesses of MAE-based ECG model include the memory requirements of the ViT itself and the cost of pre-training, although data collection is easy because of self-supervised learning using unlabeled ECG data. Comprehensive evaluation from above, we emphasize that our proposed MAE for ECG can effectively train high-capacity ViT and achieve high performance.

The ECG data we used for pretraining ECG models were collected from academic medical centres in Japan. Although detailed patient characteristic information about the patients who participated in this study was difficult to ascertain, the ECG data were mostly from Asian and Japanese patients, which suggests a lack of racial diversity. To emphasize the usefulness of the MAE-based ECG model, we validated the performance of that model on PTB-XL, which is one of the largest open access benchmark datasets. The MAE-based ECG model figure a higher macro-AUC for all statements, diagnostic statements, and rhythm statements compared with the benchmark scores. In the analysis, we did not use data augmentation or transformations to demonstrate pure model performance, and used 10-second, 500 Hz ECG data because of the structure of the MAE-based ECG model. Although PTB-XL is one of the largest open access benchmark datasets, it is relatively small compared with non-public datasets used in previous studies [31, 32]. However, the MAE-based ECG model performed admirably, even on this benchmark dataset. Thus, the high performance of the high-capability ViT on such a relatively small dataset like PTB-XL reemphasizes the usefulness of the proposed MAE-based pretraining on ECG data. By contrast, the MAE-based ECG model did not outperform the benchmark scores on the form statements, which might represent a challenging task, and the number of training data might not be sufficient to fine-tune ViT for such a challenging task.

The pragmatic standpoint of our proposed method should be noted. Our proposed method provides one option for improving performance, which is a major barrier to the implementation of DNN algorithms in the medical field. DNN algorithms based on limited medical data perform well on internally validated data, but have difficulty in maintaining performance on externally validated data. The MAE-based ECG model effectively used limited ECG data, thereby achieving high performance for external validation datasets, and maintained higher performance than the existing methods on internal test data and our pretrained ECG model also performed well on the benchmark dataset. While it is expected that models will be developed to enable a variety of ECG-based tasks in the future, acquiring large amounts of training data and maintaining performance are important challenges. To address this challenge, other researchers may be able to use our trained ECG model as one ECG general model to facilitate the implementation of ECG analysis algorithms in the medical field. Additionally, high-capability ViTs have recently become the foundation for multi-modal models [33–35], and we believe that the ability to use such data-hungry models for medical data could be an important step towards creating multi-modal DNN algorithms in the medical field, but we did not explore this task in this study, and further research is warranted.

The present study has the following limitations. First, we collected the ECG-echocardiographic data from multiple facilities. However, we were unable to gather detailed patient information. Consequently, it is difficult to distinguish whether the patients were all outpatients or if the study included inpatients as well. Furthermore, we have not been able to conduct a thorough examination regarding patients who already had arrhythmias or cardiac shunts. This lack of detailed patient characteristic information (such as vital signs, medical history, medications, and heart failure status) may result in bias in the development of ECG models. Second, although echocardiographic techniques are expected to vary among examiners and facilities, we did not perform central analysis in this study. Therefore, it is difficult to address variations in echocardiographic findings. Third, as we mentioned, the participating institutions in this study were only Japanese facilities, and the racial difference cannot be discussed. Fourth, in the ECG model pretraining with MAE, hyperparameters such as patch size, masking ratio, masking strategy, batch size, learning rate, and epoch are being referenced from previous studies for those that are possible. However, due to the significant cost of training, detailed hyperparameter exploration has not been conducted, and this study does not demonstrate the optimal hyperparameters. Lastly, we created a DNN algorithm for limited labels and limited tasks, but we cannot guarantee the performance of the model for other ECG-based tasks which we did not consider in this study.

## Conclusions

We developed high performance MAE-based ECG analysis models using limited data. The performance of these models was also found to be high depending on the model capacity and the amount of training data. The MAE-based pretrained ECG analysis model showed useful performance on the benchmark dataset PTB-XL. MAE-based pretrained ECG analysis models could be useful for various ECG-based tasks.

## Supporting information

S1 File (ZIP)

## Abbreviations

UTokyo: The University of Tokyo Hospital
Mitsui: Mitsui Memorial Hospital
Asahi: Asahi General Hospital
Sakakibara: Sakakibara Heart Institute
Jichi: Jichi Medical University Saitama Medical Center
TokyoBay: Tokyo Bay Urayasu Ichikawa Medical Center
JR: JR Tokyo General Hospital
NTT: NTT Medical Center Tokyo
