# Supplementary material for: Applying masked autoencoder-based self-supervised learning for high-capability vision transformers of electrocardiographies
Source: PLoS One. 2024 Aug 14;19(8):e0307978. doi: 10.1371/journal.pone.0307978 (PMC11324121; doi:10.1371/journal.pone.0307978)
Supplement: S1 File — (ZIP) [file pone.0307978.s001.zip › Supporting information.docx]

**Supporting information**

**S1 Fig. Qualitative sense of ECG reconstruction in Random Masking Strategy.**

We qualitatively assessed the validity of the ECGs reconstructed by the physician. The figure shows the example of random masking strategy. As shown in the figures, the reconstructions maintained the relationships among the major ECG components, such as the P wave, QRS wave, and T wave. Additionally, there was no significant discrepancy in the information between the II, III, and aVF leads (with information about the lower wall of the heart) and the V1, V2, V3, V4, and V5 leads (with information about the anterior wall), which indicate the position information of the ECG.

**S2 Fig. Example of Grid-based Masking Strategy.**

A method that masks patches at regular intervals. The positions of the patches are fixed. Each patch corresponds to 0.5 seconds of the electrocardiogram, similar to random masking.

**S3 Fig. Example of Random masking per Lead Strategy.**

A method where masking is done randomly for each lead. With a masking ratio of 75%, in a 12-lead ECG, nine leads are randomly masked, leaving the information of three leads unmasked

**S1 Table. Patient characteristics in the large dataset from three institutions.**

|  | Training dataset  (n = 91,521, 70.1%) | Validation dataset  (n = 19,733, 15.1%) | Test dataset  (n = 19,250, 14.8%) | *P*-value |
| --- | --- | --- | --- | --- |
| Participant characteristics |  |  |  |  |
| Age, (years) | 68.0 ± 15.0 | 68.0 ± 15.2 | 68.1 ± 15.1 | 0.457 |
| Age, groups |  |  |  |  |
| ≤ 30 | 2,183 (2.4) | 539 (2.7) | 519 (2.7) | <0.001 |
| 30 – 50 | 10,264 (11.2) | 2,142 (10.9) | 2,000 (10.4) |  |
| 50 – 70 | 33,090 (36.2) | 7,003 (35.5) | 6,972 (36.2) |  |
| 70 – 90 | 44,006 (48.1) | 9,653 (48.9) | 9,328 (48.5) |  |
| > 90 | 1,978 (2.2) | 396 (2.0) | 431 (2.2) |  |
| Sex |  | n = 19,730 | n = 19,249 |  |
| Female, n (%) | 36,437 (39.8) | 7,865 (39.9) | 7,688 (39.9) | 0.945 |
| male, n (%) | 55,084 (60.2) | 11,865 (60.1) | 11,561 (60.1) |  |
| Body height, | 161.2 ± 14.9(n = 82,577) | 161.5 ± 19.0(n = 17,756) | 161.1 ± 14.5(n = 17,351) | 0.082 |
| cm ± SD |  |  |  |  |
| Body weight, | 60.8 ± 14.6(n = 83,329) | 60.8 ± 13.9(n = 17,939) | 60.7 ± 14.7(n = 17,525) | 0.624 |
| kg ± SD |  |  |  |  |
| Mean EF, | 60.8 ± 13.6(n = 88,480) | 61.0 ± 13.5(n = 19,091) | 60.7 ±1 3.8(n = 18,632) | 0.103 |
| (%) ± SD |  |  |  |  |
| LVSD, n (%) | 7,881 (n = 88,480, 8.9) | 1,601 (n = 19,091, 8.4) | 1,722 (n = 18,632, 9.2) | 0.012 |
| Institution |  |  |  |  |
| UTokyo | 26,698 (29.2) | 5,787 (29.3) | 5,760 (29.9) | 0.350 |
| Mitsui | 33,848 (37.0) | 7,278 (36.9) | 7,064 (36.7) |  |
| Asahi | 30,975 (33.8) | 6,668 (33.8) | 6,426 (33.4) |  |

Data are expressed as mean ± standard deviation or number (percentage).

Pearson’s chi-square test was used for categorical variables, the Student’s *t*-test was used for normally distributed continuous variables, and the Mann–Whitney U test was used for non-normally distributed continuous variables.

EF, ejection fraction; and LVSD, left ventricular systolic dysfunction. UTokyo, The University of Tokyo Hospital; Mitsui, Mitsui Memorial Hospital; Asahi, Asahi General Hospital.

**S2 Table. The difference in LVSD detection performance of the MAE-based ECG model depends on the masking ratio.**

|  | Masking ratio 50% | Masking ratio 75% | Masking ratio 90% |
| --- | --- | --- | --- |
| AUROC | 0.948  (95% CI: 0.937–0.958) | 0.961  (95% CI: 0.950–0.970) | 0.959  (95% CI: 0.947–0.968) |

We trained the ViT-Large model using the internal cohort’s training and validation data, and assessed its LVSD detection performance on the internal cohort's test data. We utilized mask ratio of 50%, 75%, and 90%, with the masked regions chosen randomly.

|  | Random Masking | Grid-based | Random per Lead |
| --- | --- | --- | --- |
| AUROC | 0.961  (95% CI: 0.950–0.970) | 0.949  (95% CI: 0.940–0.958) | 0.955  (95% CI: 0.944–0.963) |

**S3 Table. The difference in LVSD detection performance of the MAE-based ECG model depends on the masking strategies.**

We trained the ViT-Large model using the internal cohort’s training and validation data and evaluated its LVSD detection performance on the internal cohort's test data. We applied three masking strategies: random, grid-based, and random per lead, with a masking ratio of 75%.
