# Supplementary figures and images for: Applying masked autoencoder-based self-supervised learning for high-capability vision transformers of electrocardiographies
Source: PLoS One. 2024 Aug 14;19(8):e0307978. doi: 10.1371/journal.pone.0307978 (PMC11324121; doi:10.1371/journal.pone.0307978)

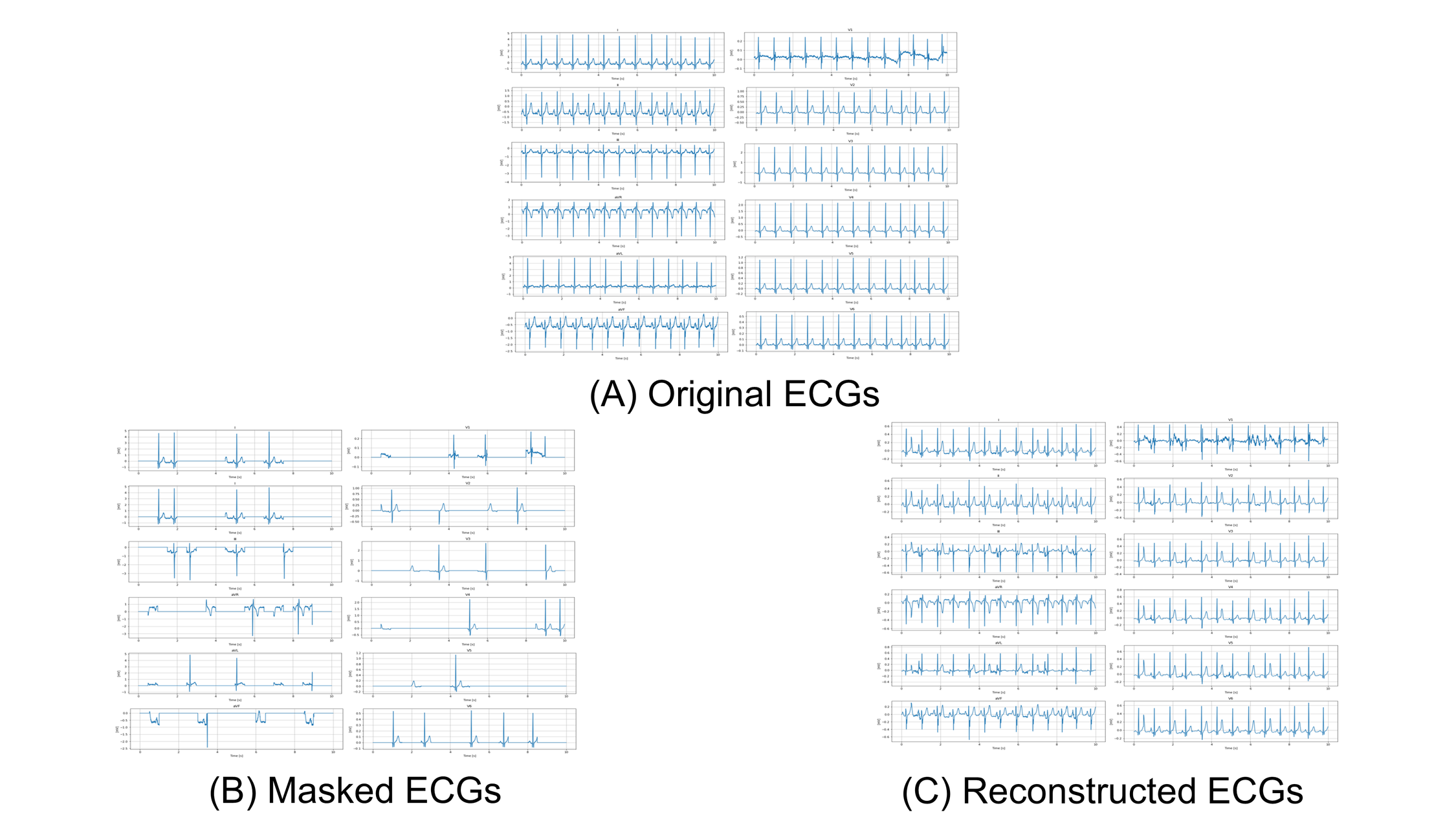

Supplement: S1 File — (ZIP) [file pone.0307978.s001.zip › S1 Fig.tif]

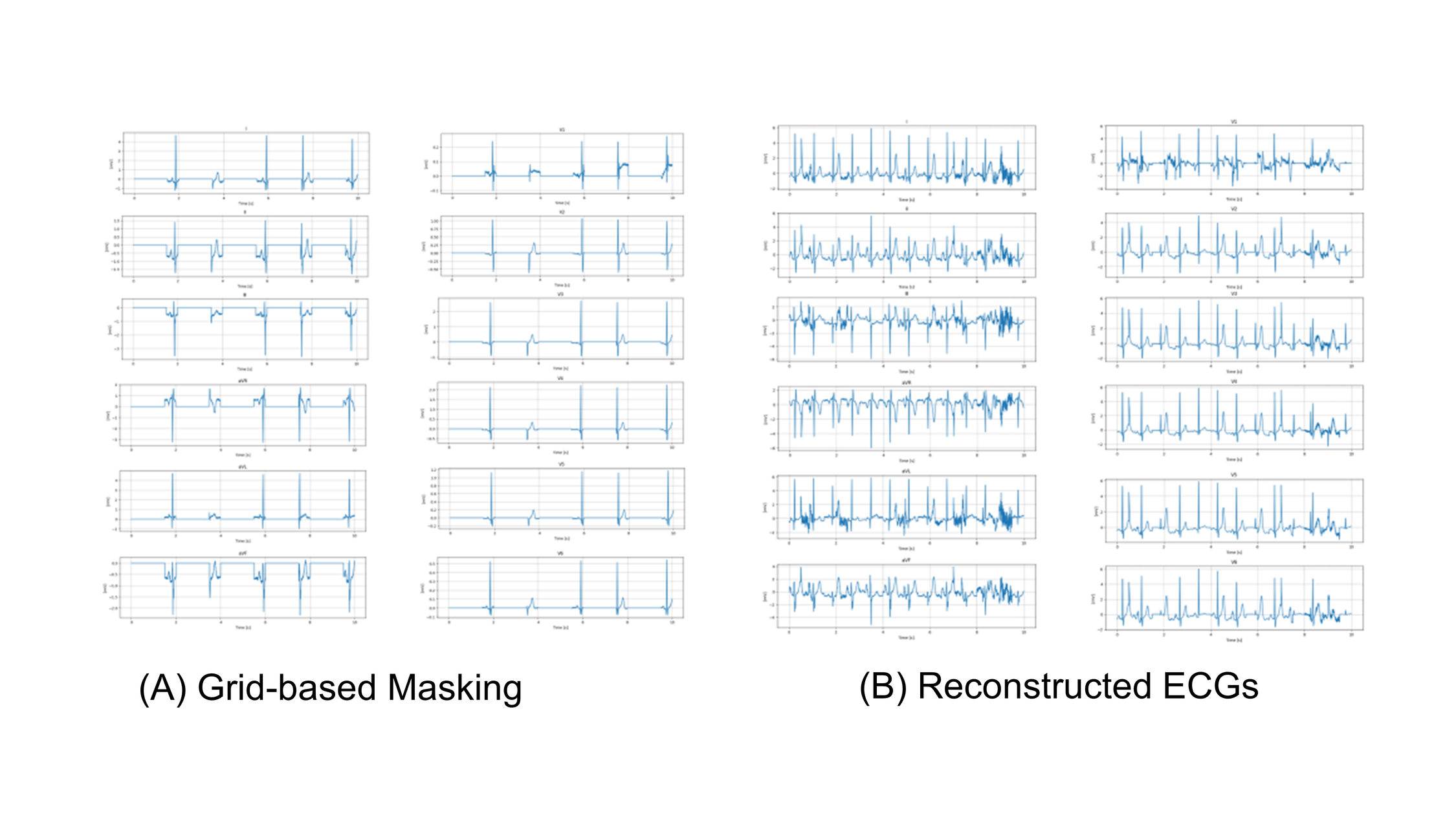

Supplement: S1 File — (ZIP) [file pone.0307978.s001.zip › S2 Fig.tif]

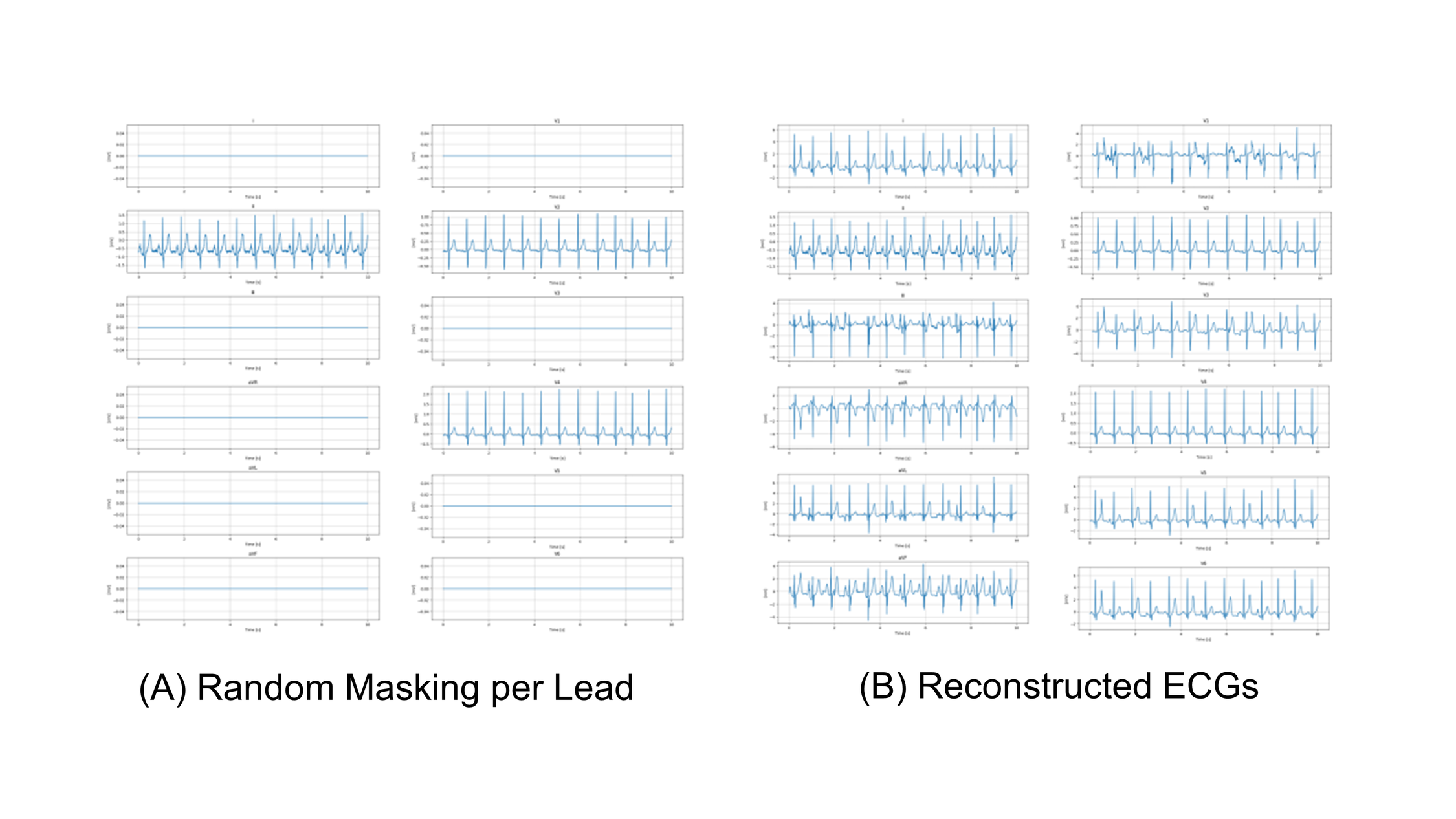

Supplement: S1 File — (ZIP) [file pone.0307978.s001.zip › S3 Fig.tif]
